# Editorial: Novel developments for promoting health through microbiota modulation

**DOI:** 10.3389/fnut.2023.1331665

**Published:** 2023-11-17

**Authors:** Carlos Gómez-Gallego, Hani El-Nezami

**Affiliations:** ^1^Institute of Public Health and Clinical Nutrition, School of Medicine, Faculty of Health Sciences, University of Eastern Finland, Kuopio, Finland; ^2^School of Biological Science, Faculty of Science, The University of Hong Kong, Hong Kong, China

**Keywords:** probiotics, prebiotics, synbiotics, fecal transplants, obesity, type 2 diabetes, hyperuricemia, constipation

The human microbiota is a vast and complex ecosystem of microorganisms that play a pivotal role in health, influencing many important bodily functions, including digestion, immunity, and metabolism (1). Recent evidence has identified associations of differences in microbiota composition with certain conditions and diseases, for example, in late-onset breast milk jaundice (Guo Q. et al.), increasing the interest in microbiota modulation as a potential treatment of certain conditions and diseases. However, findings from microbiota-related studies are ambiguous and sometimes difficult to reproduce, while defining what constitutes a “healthy microbiota” still remains a challenge (2). Interventions to alter microbiota composition have been studied for decades, ranging from dietary changes to fecal transplants. However, continuing to explore novel and more effective interventions could help to combat the rise of non-communicable diseases worldwide.

Our Research Topic contributes to the advancement of the understanding of microbiota-related interventions and their impact on human health. The 15 publications collected explore the therapeutic potential of microbiota modulation using different interventions such as probiotics, synbiotics, dietary supplements and dietary interventions in improving immune system, obesity, type 2 diabetes (T2D) and gastrointestinal disorders. These studies highlight the emerging interest to develop strategies based on microbiota modulation in disease management and, due to the individual variation on human microbiota, underscore the importance of personalized approaches to promote overall health.

Fecal microbiota transplants have been studied since the 1980s with variying level of success. In the case of the irritable bowel syndrome (IBS), a meta-analysis conducted by Zhao et al. revealed that the success of these interventions is highly dependent on several factors such as dosage, frequency, delivery method and preparation method of donor stool, as well as differences in the microbiota of the selected donors. In particular, direct delivery to the gastrointestinal tract using more invasive routes like gastroscopy or colonoscopy is more likely to lead to significant improvements of IBS symptoms when compared with oral administration. This meta-analysis highlights the need for more standardized intervention processes for the use of fecal transplants when treating specific diseases like IBS.

Microbiota modulation represents a promising strategy for improving health outcomes and managing metabolic disorders, particularly T2D and obesity. In a systematic review from Paul et al., the authors delve into the potential role of microbiome therapies in ameliorating biomarkers of inflammation and oxidative stress in patients living with T2D. Compared with individual probiotics, synbiotics, and other prebiotics, multi-strain probiotic combinations and prebiotics like resistant dextrin and inulin seem to be more effective in reducing inflammation and oxidative stress associated with T2D and could contribute to their management. This reduction in oxidative stress and inflammation may also alleviate liver damage (Al-Najjar et al.).

Positive effects of a multi-strain probiotic blend tested on children living with obesity have also been demonstrated, emphasizing how probiotic combinations can be used to reshape the gut microbiota and improve lipid metabolism and reduce body weight (Chen et al.). An intervention conducted by Yildrim et al. in children living with obesity, which evaluated the use of multi-strain synbiotic supplementation in combination with a standard diet and increased physical activity, observed significant improvements in anthropometric measurements, including body weight, BMI, and waist circumference. Both studies highlight the synergy between dietary modifications and synbiotics during weight loss, and emphasize the potential role of multi-strain probiotics and synbiotics as part of an effective weight-loss strategy in pediatric obesity.

All together, these findings underscore the combined effect of multi-strain probiotics and synbiotics to modulate microbiota composition and activity in both children and adults, with the aim of improving health and enhancing disease management.

However, it is not only the combination of different strains that have potential applications for promoting health. The consumption of particular single probiotic strains may confer benefits for the management of specific conditions, as was recognized in guidelines recently published by the World Gastroenterology Organization.^1^ In a study conducted by Guo Y. et al. in ICR mice, *Latilactobacillus sakei* Furu 2019, when administered alone or as a synbiotic with stachyose, significantly alleviates constipation, potentially through its regulation of inflammation, neurotransmitters, hormones and the gut microbiota. *Brevibacillus laterosporus* BL1 might be another promising probiotic for weight management. Prophylactic treatment of C57BL/6 mice with this strain led to reduced body weight gain, decreased fat mass, improved lipid profiles, and enhanced brown adipose tissue thermogenesis, while also positively modulating the gut microbiota (Weng et al.). *Lactiplantibacillus pentosus* P2020 was evaluated as potential preventive strategy for hyperuricemia (Wang et al.) using Kunming mice as an animal model. *Lactiplantibacillus pentosus* P2020 administration was able to reduce serum uric acid levels and mitigate renal inflammation, potentially by regulating inflammatory pathways, enhancing uric acid excretion, and improving intestinal barrier function. In germ-free Fischer 344 rats inoculated with infant fecal microbiota, Rocha Martin et al. studied how *Cutibacterium avidum* P279 can modulate microbiota composition and activity, reducing H_2_-producing bacteria with a reduction in symptoms of infant colic. However, the efficacy of these probiotics must still be demonstrated in humans.

Various nutritional and dietary interventions have also been explored to understand their impact on microbiota modulation as an approach to manage different health conditions. Lakshmanan et al. studied the impact of a formulated fruit and vegetable supplement on gut microbiota composition and antioxidant capacity. Supplementation led to changes in microbiota composition and activity, with increased *Faecalibacterium* abundance and decreased *Ruminococcus*, along with higher production of short-chain fatty acids (SCFAs). These changes could potentially reduce pro-inflammatory responses and increase antioxidant capacity in healthy adults. In interleukin-10 knockout mice, dietary eggshell membrane supplementation demonstrated significant improvements in survival rates and mitigated gut dysbiosis, suggesting that this supplement might be explored in future studies as a dietary intervention for inflammatory bowel disease in humans (Yang et al.). Interestingly, Beaumont et al. proposed that specific amino acids my be used as a novel type of prebiotics due to their selective use by intestinal bacteria and the positive impact on host health, or as synbiotics when combined with specific probiotics. Intermittent fasting (IF) is a dietary approach that has gained popularity in recent years. Mohr et al. evaluated IF as an alternative to daily caloric restriction in adults with overweight and obesity. The study compared one-day and two-day IF protocols combined with protein pacing. While both IF groups exhibited differences in fecal microbiome and plasma metabolome, the two-day IF group displayed distinct metabolic changes in plasma, such as increased levels of specific metabolites, including trimethylamine oxide, levulinic acid, 3-aminobutyric acid, citrate, isocitrate, and glucuronic acid. This research highlights the potential of IF to beneficially modulate microbiota composition and activity and suggests that varying fasting durations may be of interest for specific dietary intervention strategies.

Changes in the microbiome can also be used as novel diagnostic tools of specific diseases, as discussed by Hardinsyah et al.. They hypothesized that salivary microbiome could be used as a diagnostic indicator for T2D. Although the relationship between the salivary microbiome and T2D is complex, with large individual variation in bacterial profiles, metabolic pathways, and oral health status, the authors highlight the potential of salivary microbiome profiling as a rapid, non-invasive test for detecting T2D. As microorganisms can respond quickly to changes in the human body, there is promise in the use of them as biological indicators, for both rapid diagnosis and monitoring the effectiveness of therapeutic interventions.

Microbiota modulation, as highlighted in our Research Topic, has great potential for improving health. It can positively impact conditions such as obesity, hyperuricemia, constipation, and T2D. The use of fecal microbiota transplants, probiotics, prebiotics, synbiotics, dietary supplements, and dietary interventions demonstrates the versatility of this approach. Future research should focus on refining specific microbial targets, personalized interventions, and addressing the interplay between diet, microbiota, and health. Additionally, exploringthe microbiome as diagnostic tool, and harnessing microbiota modulation in other less explored areas like cardiovascular health and mental wellbeing, are exciting future possibilities. These studies contribute to set the basis for innovative applications and open the door to new frontiers in microbiota-related research.

## Author contributions

CG-G: Writing—original draft, Writing—review & editing. HE-N: Writing—original draft, Writing—review & editing.

## Footnotes

^1^WGO. Available online at: https://www.worldgastroenterology.org (accessed October 31, 2023).

## References

[1] Hou K Wu Z-X Chen X-Y Wang J-Q Zhang D Xiao C al. Microbiota in health and diseases. Sig Transduct Target Ther. (2022) 7:1–28. 10.1038/s41392-022-00974-4 PMC9034083 35461318

[2] Shanahan F Ghosh TS O'Toole PW. The healthy microbiome-what is the definition of a healthy gut microbiome? Gastroenterology. (2021) 160:483–94. 10.1053/j.gastro.2020.09.057 33253682

